# Accessing Take-Home Naloxone in British Columbia and the role of community pharmacies: Results from the analysis of administrative data

**DOI:** 10.1371/journal.pone.0238618

**Published:** 2020-09-11

**Authors:** Amina Moustaqim-Barrette, Kristi Papamihali, Zahra Mamdani, Sierra Williams, Jane A. Buxton

**Affiliations:** 1 BC Centre for Disease Control, Vancouver, BC, Canada; 2 School of Population and Public Health, University of British Columbia, Vancouver, BC, Canada; University of Manitoba, CANADA

## Abstract

**Introduction:**

British Columbia’s (BC) Take-Home Naloxone (THN) program provides naloxone to bystanders for use in cases of suspected opioid overdose. This study seeks to provide trends and analysis from the provincial BC THN program since inception in 2012 to the end of 2018.

**Materials and methods:**

BC THN shipment and distribution records from 2012–2018 were retrieved. Frequency distributions were used to describe characteristics of individuals accessing the program. To evaluate correlates of distribution after the addition of hundreds of pharmacy distribution sites, an analytic sample was limited to records from 2018, and multivariate logistic regression was used to evaluate correlates of collecting naloxone at a pharmacy site.

**Results:**

Since program inception to the end of 2018, there were 398,167 naloxone kits shipped to distribution sites, 149,999 kits reported distributed, and 40,903 kits reported used to reverse an overdose in BC. There was a significant increasing trend in the number of naloxone kits used to reverse an overdose over time (p<0.01), and more than 90% of kits that were reported used were distributed to persons at risk of an overdose. Individuals not personally at risk of overdose had higher odds of collecting naloxone at a pharmacy site, compared to other community sites (including harm reduction supply distribution sites, peer led organizations, drop-in centers, and supportive housing sites) (Adjusted Odds Ratio (AOR): 2.69; 95% CI: 2.50–2.90).

**Conclusions:**

This study documents thousands of opioid overdose reversals facilitated through the BC THN program. While those at highest risk of overdose may preferentially access naloxone through community sites, naloxone distribution through pharmacies has allowed the BC THN program to expand dramatically, increasing naloxone availability through longer opening hours on evenings and weekends. and in rural and remote regions. A diversity of naloxone distribution sites and strategies is crucial to prevent rising opioid overdose deaths.

## Introduction

The province of British Columbia (BC) currently finds itself amid dual public health emergencies–the first declared on April 14, 2016 related to an unprecedented increase in opioid overdose deaths [1], and the second declared on March 17, 2020 [2], related to the ongoing pandemic of coronavirus disease 2019 (COVID‑19) [3].

Between January 2016 and December 2018, over 11,000 people died of suspected opioid overdose in Canada–over 4500 of these in BC [4]. During this time, the potent synthetic opioid fentanyl and its analogues were detected in over 80% of illicit drug toxicity deaths in the province of British Columbia [5]. Distribution of naloxone, a μ-opioid receptor antagonist drug, has been identified as a key emergency measure to prevent opioid-related overdoses and overdose deaths [6]. When used appropriately, naloxone is effective at reversing the symptoms of opioid toxicity and life-threatening respiratory depression [7,8].

Since August 31, 2012, the British Columbia Centre for Disease Control (BCCDC) has overseen a centralized provincial Take-Home Naloxone (THN) program for the province of British Columbia (BC), with support from the BC Ministry of Health. As one of the first of its kind in Canada [9], the BC THN program aims to train individuals in overdose recognition and response as well as provide naloxone kits free of charge to any person at risk of experiencing an opioid overdose. The program was expanded to those likely to witness an opioid overdose, such as family and friends, in September 2016. Naloxone kits include a carrying case, non-latex gloves, alcohol swabs, a face shield with a one-way valve and large impermeable area to protect the responder from respiratory secretions when providing rescue breaths, three safety syringes, three 0.4 mg/mL naloxone ampoules (each with an ampoule breaker), a naloxone overdose response information or ‘administration form’, and an instructional overdose response infographic. An online training application was also developed to ensure brief standardized training was available to individuals at risk of experiencing or witnessing an overdose prior to receiving THN kit [10]. Training modules also serve as a brief refresher for pharmacists and pharmacy staff. Training is delivered through an online course, approximately 10 minutes in length, which offers video tutorials, knowledge tests, and a certificate of completion available online and on mobile [10]. More comprehensive training videos and resources are also available [10].

When developing the program, the BCCDC first prioritized making naloxone available through harm reduction supply distribution sites and community organizations in areas where there was high prevalence of overdose, with the goal of meeting individuals where they were at and reducing accessibility barriers to those at highest risk of overdose. In particular, harm reduction supply distribution sites encompass a variety of low-barrier services in which clients are able to obtain safer sex and drug use supplies [11]. Other low-barrier community-based include peer-led and non-governmental organizations, hospitals and health centres, treatment centres, supportive housing sites, hospitals, and emergency departments [12]. BC THN kits are also made available upon release from provincial correctional facilities [13].

Several regulatory changes occurred which led to the expansion of the BC THN program. In March 2016, Health Canada removed naloxone from the Prescription Drug List and the College of Pharmacists of BC made it available as a Schedule II drug (behind the counter) [12]. In September 2016, the College of Pharmacists of BC changed the status of naloxone for emergency use to ‘unscheduled’, making it available outside of pharmacies [14]. Furthermore, the BC THN program removed the requirement to report individual naloxone kit IDs and/or patient names in October 2016 [15].

In December 2017, the BC THN program officially expanded to select community pharmacies to improve BC THN access across the province. Pharmacies are available widely, including in remote and hard-to-reach communities, with longer hours (including weekends and evenings), and they may be more acceptable to clients less likely to frequent harm reduction supply distribution sites [16]. The program partnered with pharmacy wholesalers, banners and chains to onboard pharmacies through centralized distribution models initially, later reaching stand-alone pharmacies in remote areas with limited access to harm reduction supply distribution sites. By the end of 2018, there were 1,448 active take-home naloxone distribution sites across BC [17], including 562 community pharmacy sites [18]—representing 40.5% of all pharmacies [19].

Understanding correlates of take-home naloxone distribution and access is crucial for planning and targeting services aimed at curbing opioid overdoses. While analyses from the BCCDC shows that the BC THN program, combined with other harm reduction interventions, has averted thousands of opioid overdose deaths [20, 21], there is still relatively little data in the published literature examining community reach and barriers to access of naloxone distribution programs in Canada. Furthermore, preliminary evidence from the BC Coroners Service suggests a spike in drug toxicity deaths corresponding with COVID-19 physical distancing measures and changes to the illicit drug supply; the Coroners Service reported a 130% increase in drug toxicity deaths in June 2020 compared to June 2019 [22]. As overdose deaths continue to rise and organizations critical in the training and distribution of naloxone face reduced hours and temporary closures due to COVID-19 [23], there is a need to understand barriers and access associated with different avenues of distribution.

The current paper seeks to first provide an overview of uptake of the BC THN program since inception in August 2012 to the end of 2018, as well as correlates of naloxone distribution through pharmacy sites after the BC THN’s pharmacy expansion in late 2017. Findings may help inform optimization of take-home naloxone programs across jurisdictions.

## Materials and methods

### 2.1 Ethics

This study analysed anonymous administrative data. Ethics approval was obtained from the University of British Columbia Behavioural Ethics Board (H12-02557).

### 2.2 Data sources

The BC THN program draws on two key sources of data to monitor and evaluate naloxone distribution and use across BC. Firstly, ‘supply order forms’ were used internally at the BCCDC to track the number of kits shipped, to participating distribution sites [9].

Second, ‘distribution records’ were used by naloxone distribution sites to track characteristics of individuals who receive a naloxone kit [10]. No personal identifiers were collected, and sites were asked to return completed distribution records to the BCCDC on a monthly basis. Since program inception, distribution records have included site-specific information (site’s unique ID, city, and the date the kit was distributed to an individual) completed by on-site staff, as well as a ‘site type’ identifier (corrections facilities, pharmacy, post-secondary, and other (housing sites, treatment centres, non-governmental and peer-led organizations). Individuals collecting a kit were asked to self-report their gender, their age range, and whether they are collecting a first or replacement kit (due to the previous kit being used, lost, stolen, expired or confiscated).

Following the unscheduling of naloxone and THN program expansion in 2017 to individuals not personally at risk of an overdose, distribution record forms were amended to include self-reported risk of overdose i.e. personally at risk of overdose or at risk of witnessing an overdose (i.e. friends and family of someone at risk of overdose). Before this date, all kit recipients were assumed to be personally at risk of overdose.

Distribution records often experience a delay in return and entry due to two reasons: 1) a delay and general decrease in submission of records from distribution sites and, 2) a lag in manual entry of records at the BCCDC. Together, we estimate the return window to be on average between three to six months.

### 2.3 Study variables

Supply order forms were geographically coded by health authority (Vancouver Coastal Health, Fraser Health, Island Health, Interior Health, and Northern Health) and used to provide the number of naloxone kits shipped to distribution sites over time.

Distribution records were the primary data source used in this study. From these records, site-specific geographical information was used to classify observations by Health Authority (Vancouver Coastal Health, Fraser Health, Island Health, Interior Health, and Northern Health). Records were also used to classify records by age group (<19, 19–30, 31–60, >60, Unknown), gender (Male, Female, Other (Trans, gender non-conforming), Unknown), reason for collecting a kit (1^st^ kit, replacement kit–used, replacement kit–other (previous kit lost, stolen, confiscated or expired), site type (corrections facility, pharmacy, post-secondary institution, other (housing sites, treatment centres, non-governmental and peer-led organization), and 'eligibility for collecting a naloxone kit (not personally at risk of overdose, peronlly at risk of overdose, unknown).

### 2.4 Analysis plan

This study includes findings from distribution records and supply order forms across BC from program inception on August 31^st^, 2012 to December 31^st^, 2018. To evaluate the effect of the THN program’s expansion into community pharmacies, a separate analytic sample was created which limits distribution records to those returned between January 1^st^, 2018 and December 31^st^, 2018. Data for this study were retrieved and analyses conducted in January 2020. All analyses were conducted using R version 3.5.3 [24].

Frequency distributions were used to describe characteristics of individuals accessing the BC THN program from 2012 to the end of 2018, as well as to compare shipment and distribution data. A chi-squared test for trend (Cochran-Armitage test) was used to evaluate differences in the proportion of naloxone kits used to respond to an opioid overdose over time. Frequency distributions were subsequently used to examine the separate analytic sample, limited to 2018 distribution records. In this sample, all non-pharmacy sites were grouped as ‘other’ sites to compare with pharmacy distribution. Multivariate logistic regression was used to examine factors associated with distribution site type.

The model building strategy described by Hosmer and Lemeshow [25], and further operationalized by Zhang [26], was employed. All available covariates were included for bivariable analysis to explore trends and examine distributions by the outcome variable. Covariates were cross-tabulated with the outcome variable and those with a p-value < 0.25 and were deemed to be conceptually relevant as indicated in the literature, were included for further multivariate analysis. As per the purposeful selection model building strategy, variables which did not meet the p-value cut-off but were known to be conceptually relevant were included in further analyses. Adjusted odds ratios (AOR), 95% confidence intervals (CI), and p-values are reported. P-values less than 0.05 were considered statistically significant.

## Results

### 3.1 Take-home naloxone program uptake

**Fig 1:**
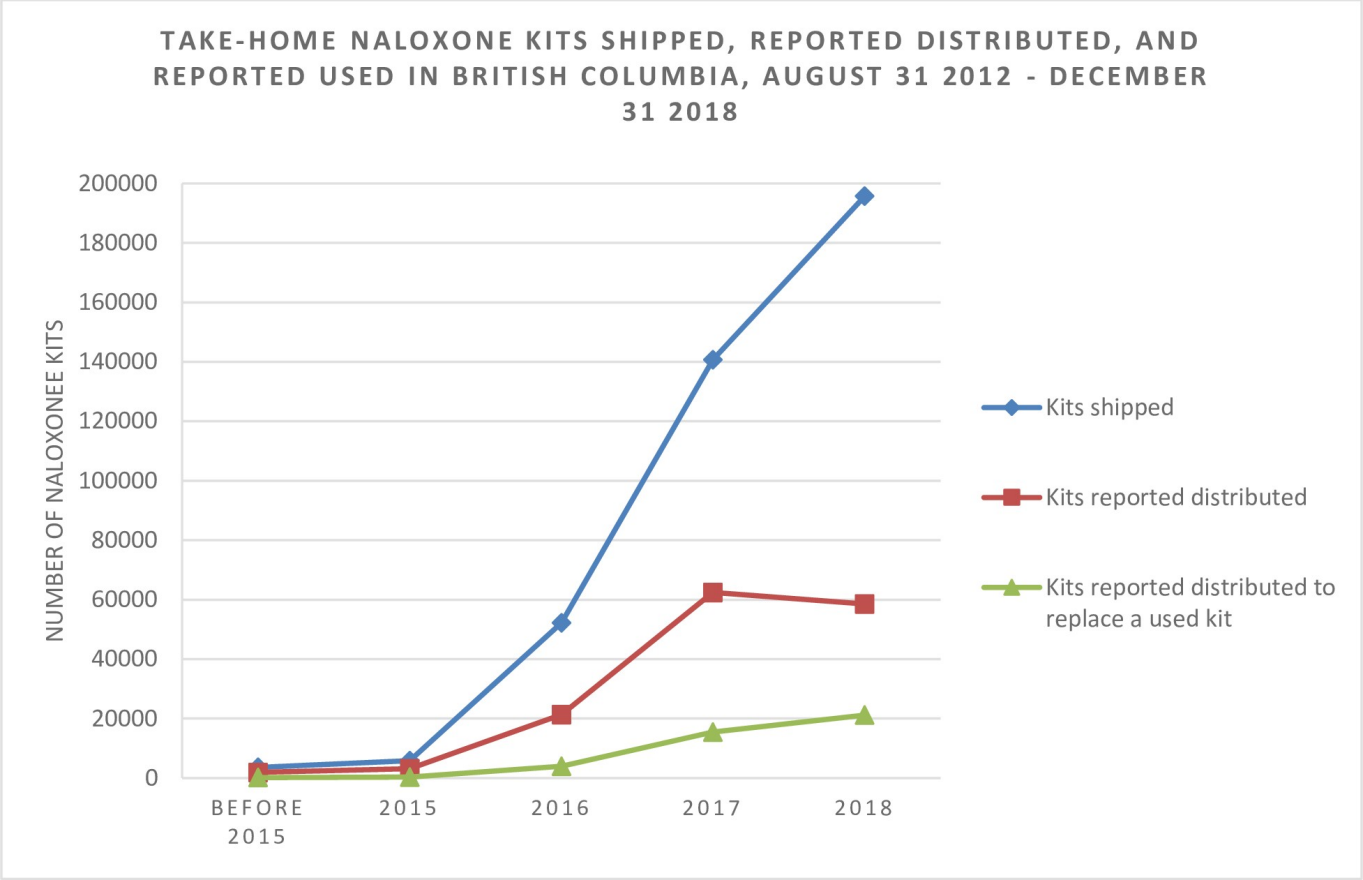
Graphical representation of the number of take-home naloxone (THN) kits shipped, reported distributed, and reported used from THN program inception on August 31st, 2012 to December 31st, 2018.

Fig 1 and Table 1 provide a summary of naloxone kits shipped, reported distributed, and reported used to reverse an overdose from the BC THN program, from August 31^st^, 2012 to December 31^st^, 2018. During this time period, a total of 398,167 kits were shipped and 149,999 naloxone kits were reported distributed, according to records returned. Data show a consistent increase in kits shipped, however the number of kits reported distributed increased through 2017 but dropped in 2018 (Fig 1).

**Table 1:** Number of take-home naloxone kits shipped, reported distributed, and reported used, from program inception on August 31^st^ 2012 to December 31^st^ 2018.

	Before 2015	2015	2016	2017	2018	Total
	n (%)	n (%)	n (%)	n (%)	n (%)	n (%)
**Kits shipped**	3575 (0.9%)	5886 (1.5%)	52262 (13.1%)	140748 (35.4%)	195696 (49.2%)	398167 (100.0%)
**Kits reported distributed**	1921 (1.3%)	3153 (2.1%)	21293 (14.4%)	62445 (42.4%)	58547 (39.7%)	147359 (100.0%)
**Kits reported distributed to replace a used kit**	168 (0.4%)	397 (1.0%)	3941 (9.6%)	15519 (37.9%)	21172 (51.1%)	41197 (100.0%)

A total of 41,197 kits (27.5% of those distributed) were reported to have been obtained to replace a kit used to respond to an opioid overdose. As a proportion of naloxone kits reported distributed, naloxone kits reported used to reverse an overdose increased steadily over time, from 8.7% in the pre-2015 period (168/1921), to 12.6% in 2015 (397/3153), 18.5% in 2016 (3941/21,293), 24.9% in 2017 (15,519/62,445), and 36.2% in 2018 (21,172/58,547). A chi-squared test for trend in proportions suggests a significant increasing trend (p <0.01).

Table 2 presents summary characteristics of BC THN distribution records, from program inception to the end of 2018. For all variables, missing information was categorized as ‘unknown’. Overall, 24,890 kits (16.6%) were distributed to replace a lost, stolen, confiscated or expired kit. The majority of THN distribution records were received in 2017 (42.2%) and 2018 (40.1%). The large majority (94.2%) of THN kits were distributed at ‘Other’ sites (including housing sites, health and treatment centres, non-governmental and peer-led organizations). The overall kit distribution rate 2012–2018 was 31.4 kits per 1000 BC population but varied between health authorities (based on average population estimates 2012–2018). The rate of kit distribution varied between health regions, from 22.4 to 50.4 kits/1,000 population, while the proportions distributed were similar between the four most populous health regions (20.8% - 26.7%), with 5.2% of kits distributed in the least populous region–see the table in S1 Appendix for details.

**Table 2:** Summary characteristics of BC Centre for Disease Control (BCCDC) naloxone distribution data, from program inception on August 31^st^ 2012 to December 31^st^ 2018.

Characteristics	1st Kit (N = 83,912)	Replacement-Used (N = 41,197)	Replacement-Other^D^ (N = 24,890)	Total (N = 149,999)	p value
	n (%)	n (%)	n (%)	n (%*)	
**Year**					< 0.001
Before 2015	1521 (79.2%)	168 (8.7%)	232 (12.1%)	1921 (1.3%)	
2015	2115 (67.1%)	397 (12.6%)	640 (20.3%)	3152 (2.1%)	
2016	14312 (66.5%)	3941 (18.3%)	3266 (15.2%)	21519 (14.4%)	
2017	36800 (58.2%)	15519 (24.5%)	10934 (17.3%)	63253 (42.2%)	
2018	29164 (48.5%)	21172 (35.2%)	9818 (16.3%)	60154 (40.1%)	
**Gender**					< 0.001
Male	30914 (47.5%)	22053 (33.9%)	12166 (18.7%)	65133 (43.4%)	
Female	31080 (57.2%)	14733 (27.1%)	8491 (15.6%)	54304 (36.2%)	
Other^A^	1029 (55.5%)	363 (19.6%)	462 (24.9%)	1854 (1.2%)	
Unknown^B^	20889 (72.8%)	4048 (14.1%)	3771 (13.1%)	28708 (19.3%)	
**Age Group**					< 0.001
Under 19	3741 (71.0%)	948 (18.0%)	582 (11.0%)	5271 (3.5%)	
19–30	22173 (53.0%)	12792 (30.6%)	6899 (16.5%)	41864 (27.9%)	
31–60	32949 (48.3%)	22469 (32.9%)	12802 (18.8%)	68220 (45.4%)	
Over 60	2798 (66.9%)	877 (21.0%)	506 (12.1%)	4181 (1.0%)	
Unknown^B^	22251 (73.0%)	4111 (13.5%)	4101 (13.5%)	30463 (20.3%)	
**Eligibility**					< 0.001
Not at risk	20747 (78.4%)	2760 (10.4%)	2955 (11.2%)	26462 (17.6%)	
At risk	22915 (36.4%)	26990 (42.9%)	13061 (20.7%)	62966 (42.0%)	
Unknown^B^	40250 (66.5%)	11447 (18.9%)	8874 (14.7%)	60571 (40.4%)	
**Health Authority**					< 0.001
Fraser	20803 (52.5%)	11544 (29.2%)	7244 (18.3%)	39591 (26.4%)	
Interior	17552 (55.9%)	8215 (26.2%)	5611 (17.9%)	31378 (20.9%)	
Island	22329 (55.7%)	12152 (30.3%)	5611 (14.0%)	40092 (26.7%)	
Northern	4916 (63.0%)	1315 (16.9%)	1569 (20.1%)	7800 (5.2%)	
Vancouver Coastal	18312 (58.8%)	7971 (25.6%)	4855 (15.6%)	31138 (20.8%)	
**Site Type**					< 0.001
Corrections	2220 (83.9%)	185 (7.0%)	242 (9.1%)	2647 (1.8%)	
Pharmacy	3532 (74.1%)	813 (17.1%)	420 (8.8%)	4765 (3.2%)	
Post-Secondary	1184 (94.6%)	21 (1.7%)	47 (3.8%)	1252 (0.8%)	
Other^C^	76971 (54.5%)	40170 (28.4%)	24181 (17.1%)	141322 (94.2%)	

*Column percentages

^A^ Other genders reported include being trans or gender non-conforming

^B^ For the age and gender variables, a high proportion of ‘unknown’ is likely due to the nature of ensuring a low-barrier THN program–some information is not always reported. For the eligibility variable, eligibility criteria was introduced in 2017, when the THN program was expanded to individuals not personally at risk of overdose. All individuals who received a kit before 2017 were considered personally at risk of overdose, and account for the majority of the ‘unknown’ category for the Eligibility variable.

^C^ Other site types include housing sites, treatment centres, non-governmental and peer-led organization

^D^ Take-home naloxone kit was replaced due to a previous kit being lost, stolen, confiscated or expired

Overall, most THN kits were distributed to individuals who identified as male (43.4%) compared to female (36.2%), other genders (1.2%), and unknown (19.3%), and to those between the ages of 19–30 (27.9%) or 31–60 (45.4%). Eligibility criteria was introduced in 2017, when the THN program was expanded to individuals not personally at risk of overdose. All individuals who received a kit before 2017 were considered personally at risk of overdose, and account for the majority of the ‘unknown’ category. For the age and gender variables, a high proportion of ‘unknown’ is likely due to the nature of ensuring a low-barrier THN program–some information is not always reported by THN distribution sites or by recipients.

### 3.2 Characteristics of individuals accessing naloxone after introduction of pharmacy sites

Table 3 presents the analytic sample characteristics after limiting naloxone distribution records to 2018, stratified by eligibility. By the end of 2018, there were 562 community pharmacy distribution sites. A breakdown of THN distributing pharmacy sites by Health Authority is provided in the table of S2 Appendix. A total of 57,524 records were included, of which 39,208 (68.2%) self-reported being personally at risk of overdose, compared to 18,316 (31.8%) of individuals who reported not being personally at risk of overdose. Of naloxone kits reported used to respond to an opioid overdose in 2018, 90.2% were kits obtained by individuals who were personally at risk of overdose. Even after the introduction of pharmacy sites across BC, a large majority (93.2%) of individuals continued to access naloxone at ‘other’ community site types, compared to pharmacy sites (6.8%). Furthermore, the majority of those who self-reported not being personally at risk of overdose accessed naloxone at a pharmacy (60.1%), while the majority of individuals personally at risk of overdose accessed naloxone at other sites (70.2%).

**Table 3:** Summary characteristics of analytic sample.

Characteristics	Not personally at risk of overdose (N = 18,316)	Personally at risk of overdose (N = 39,208)	Total (N = 57,524)	p value
	n (%)	n (%)	n (%*)	
**Gender**				< 0.001
Male	6974 (23.7%)	22484 (76.3%)	29458 (51.2%)	
Female	10315 (41.1%)	14808 (58.9%)	25123 (43.6%)	
Other^A^	482 (47.1%)	542 (52.9%)	1024 (1.8%)	
Unknown	545 (28.4%)	1374 (71.6%)	1919 (3.3%)	
**Age Group**				< 0.001
Under 19	1157 (37.9%)	1899 (62.1%)	3056 (5.3%)	
19–30	6166 (31.1%)	13650 (68.9%)	19816 (34.4%)	
31–60	9476 (30.9%)	21213 (69.1%)	30689 (53.3%)	
Over 60	869 (48.6%)	918 (51.4%)	1787 (3.1%)	
Unknown	648 (29.8%)	1528 (70.2%)	2176 (3.8%)	
**Reason for collecting a kit**				< 0.001
1st Kit	14253 (52.6%)	12827 (47.4%)	27080 (47.1%)	
Replacement- Other^B^	2019 (21.1%)	7560 (78.9%)	9579 (16.7%)	
Replacement- Used	2044 (9.8%)	18821 (90.2%)	20865 (36.3%)	
**Health Authority**				< 0.001
Fraser	3513 (21.4%)	12911 (78.6%)	16424 (28.6%)	
Interior	4439 (35.3%)	8135 (64.7%)	12574 (21.9%)	
Island	5486 (33.4%)	10927 (66.6%)	16413 (28.5%)	
Northern	1292 (33.1%)	2606 (66.9%)	3898 (6.8%)	
Vancouver Coastal	3586 (43.7%)	4629 (56.3%)	8215 (14.3%)	
**Site Type**				< 0.001
Pharmacy	2357 (60.1%)	1566 (39.9%)	3923 (6.8%)	
Other^C^	15950 (29.8%)	37638 (70.2%)	53588 (93.2%)	

Subsample taken from distribution data, stratified by eligibility (self-reported risk), from January 2018 to December 2018.

*Column percentages

^A^ Other genders reported include being a trans, and gender non-conforming

^B^ Take-home naloxone kit was replaced due to a previous kit being lost, stolen, confiscated or expired

^C^ Other site types include corrections, pharmacy, post-secondary, other (e.g housing sites, treatment centres, non-governmental and peer-led organization)

### 3.3 Correlates of collecting a THN kit through a pharmacy site

Table 4 presents the analytic sample characteristics after limiting naloxone distribution records to 2018, as well as multivariate logistic regression results (adjusted odds ratios and 95% confidence intervals) for correlates of THN kit collection at a pharmacy site, compared to other sites. In bivariate analyses, all covariates were statistically significant and conceptually relevant, and were included in the analysis.

**Table 4:** Adjusted odds ratios and 95% confidence intervals for odds of collecting a kit at pharmacy sites vs other sites.

	Summary characteristics of analytic sample			Multivariate logistic regression	
	Pharmacy (N = 3923)	Other^C^ (N = 53601)	Total (N = 57,524)		
	n (%)	n (%)	n (%*)	AOR (95% CI)	P-value
**Gender**					
Male	2053 (7.0%)	27410 (93.0%)	29458 (51.2%)	1.00	-
Female	1692 (6.7%)	23426 (93.3%)	25123 (43.6%)	0.74 (0.69–0.80)	<0.01
Other^B^	121 (11.8%)	903 (88.2%)	1024 (1.8%)	1.77 (1.44–2.18)	<0.01
Unknown	57 (3.0%)	1862 (97.0%)	1919 (3.3%)	0.39 (0.29–0.52)	<0.01
**Age**					
Under 19	197 (6.4%)	2859 (93.6%)	3056 (5.3%)	1.00	-
19–30	1466 (7.4%)	18346 (92.6%)	19816 (34.4%)	1.41 (1.21–1.66)	<0.01
31–60	1879 (6.1%)	28803 (93.9%)	30689 (53.3%)	1.19 (1.02–1.39)	0.03
Over 60	273 (15.3%)	1512 (84.7%)	1787 (3.1%)	2.43 (1.99–2.97)	<0.01
Unknown	108 (5.0%)	2068 (95.0%)	2176 (3.8%)	1.09 (0.84–1.42)	0.51
**Eligibility**					
Personally at risk of overdose	2357 (12.9%)	15950 (87.1%)	18307 (31.8%)	1.00	-
Not personally at risk of overdose	1566 (4.0%)	37638 (96.0%)	39204 (68.2%)	2.69 (2.50–2.90)	<0.01
**Reason for collecting a kit**					
1^st^ kit	2880 (10.6%)	24195 (89.4%)	27080 (47.1%)	1.00	-
Replacement–other^A^	347 (3.6%)	9232 (96.4%)	9579 (16.7%)	0.42 (0.37–0.47)	<0.01
Replacement–used	696 (3.3%)	20161 (96.7%)	20865 (36.3%)	0.46 (0.42–0.51)	<0.01
**Health Authority**					
Island	532 (3.2%)	15881 (96.8%)	16413 (28.5%)	1.00	-
Fraser	1145 (7.0%)	15279 (93.0%)	16424 (28.6%)	2.79 (2.50–3.10)	<0.01
Interior	923 (7.3%)	11651 (92.7%)	12574 (21.9%)	2.33 (2.08–2.60)	<0.01
Northern	208 (5.3%)	3690 (94.7%)	3898 (6.8%)	1.68 (1.42–1.98)	<0.01
Vancouver Coastal	1115 (13.6%)	7087 (86.4%)	8215 (14.3%)	4.07 (3.65–4.54)	<0.01

Abbreviations: AOR, Adjusted odds ratio; CI, confidence interval

*Column percentages

^A^ Take-home naloxone kit was replaced due to a previous kit being lost, stolen, confiscated or expired

^B^ Other genders reported include being trans or gender non-conforming

^C^ Other site types include corrections, pharmacy, post-secondary, other (e.g. housing sites, treatment centres, non-governmental and peer-led organizations)

After adjustment, and consistent with descriptive statistics, individuals who were not personally at risk of overdose had more than twice the odds of collecting a THN kit at a pharmacy site, compared to other sites (AOR = 2.69 [95% CI 2.50–2.90]). Individuals collecting a replacement kit were less likely to collect those kits at a pharmacy site compared to first time kit recipients (both for replacement due to previous kit being used (AOR = 0.46 [95% CI 0.42–0.51]) and for other reasons (AOR = 0.42 [95% CI 0.37–0.47])).

Females had lower odds of accessing naloxone in pharmacy (AOR = 0.74 [95% CI 0.70–0.80]), while individuals reporting an ‘other’ gender had higher odds of accessing naloxone in pharmacy (AOR = 1.77 [95% CI 1.44–2.18]), compared to males. Those between the age of 19–30 had higher odds of accessing naloxone through pharmacy compared to those under 19 (AOR = 1.41 [95% CI 1.21–1.66]), as well as those over the age of 60 (AOR = 2.43 [95% CI 1.99–2.97]). Finally, individuals in Island health authority had the lowest odds of collecting naloxone through a pharmacy site, compared with other health authorities.

## Discussion

The current study provides an overview of uptake of the BC THN program. While all Canadian provinces and territories have publicly funded THN programs, research and evaluation of these programs in the scholarly literature is limited. A recent study from Ontario describes uptake of the province’s pharmacy-dispensed naloxone kits in Ontario [27], and preliminary analyses of provincial THN programs in Ontario [28], Alberta [29], Manitoba [30], and British Columbia [15] have been published. Data from other jurisdictions are not directly comparable, due to differences in program structure, eligibility criteria, and scope [9]. Nevertheless, analyses across provincial THN programs are valuable for examining program uptake across jurisdictions. An environmental scan of provincial programs and distribution has also been conducted [9], and our group is involved in efforts to combine aggregate jurisdictional level data nationally.

The current study finds that the BC THN program expanded to a total of approximately 1,500 sites across BC by the end of December 2018, including 562 pharmacy sites (40.5% of all community pharmacies in BC) since December 2017. Overall, almost 400,000 naloxone kits were shipped and 150,000 reported distributed since the program was launched in 2012.

Our findings show that there is a significant increasing trend in the proportion of kits reported used to reverse an overdose over time. Most individuals collecting a naloxone kit self-reported being male and between the ages of 31–60. This corresponds with data from the BC Coroners Service, which has found that the group at highest risk of overdose death in BC are men between the ages of 40–59 years old, as well as those 19–39 years [31]. Importantly, our findings also indicate that the vast majority of kits reported used to reverse an overdose were distributed to persons themselves at risk of an overdose. While it is possible that individuals collect naloxone kits for others to use on them in the event they overdose, most individuals who use drugs do so around other people who use drugs. We believe our findings suggest then that people who use drugs are most likely to respond to overdoses in their communities, though further research to help elucidate this question is warranted.

After evaluating data from 2018, our analysis also finds that individuals at risk of opioid overdose were significantly less likely to collect a naloxone kit at a pharmacy site compared to community-based sites. In developing the BC THN program, the priority was to ensure a low barrier environment for individuals receiving overdose response training, collecting naloxone kits, and seeking other supports. Consistent with our findings, evidence from the literature suggests that people with lived and/or living experience of substance use may be reluctant to access pharmacies and other medical services due to past experiences of stigma or discrimination [32,33]. Nevertheless, data from this study suggests that first time naloxone kit recipients may be more likely to collect kits from pharmacy sites, suggesting that these sites are important in reaching different cohort of clients who may not access traditional distribution sites aimed at higher-risk communities.

The authors believe that these findings offer support for increased access to naloxone through pharmacy sites in BC, and highlights the importance of having a variety of distribution site types. A previous evaluation from our group suggests pharmacists are willing to be involved in the BC THN initiative, despite some initial concerns about lack of remuneration for training and dispensing. There are mixed views regarding further program growth; some pharmacy managers feel that naloxone is now fairly accessible and that there is no need for further expansion, while others feel that expansion to more pharmacy banners and chains would be beneficial [16].

Due to the COVID-19 crisis, some harm reduction services and supply distribution sites have temporarily closed or have reduced hours [23], while pharmacies remain an essential service that continue to be widely available, open, and hence accessible to distribute THN. Online training is available for those who need refresher training or to be newly trained, allowing for physical distancing during the training process. The THN kits contain non-latex gloves which responders are advised to put on before responding to an overdose and to remove carefully after responding, as well as to practice hand hygiene.

With recent changes of temporary exemptions [34] and clinical opioid prescribing guidelines [35] in relation to COVID-19, individuals may be receiving new opioid agonist therapy (OAT) prescriptions, longer prescriptions, and/or delivery of medications by pharmacies. In many cases, pharmacists have been advised to co-dispense naloxone. Future research is needed to explore changes in distribution and the role of pharmacies in providing naloxone to individuals at risk of overdose during the dual public health emergency period.

While data suggests a decline in naloxone kits reported distributed compared to kits shipped in 2018, it is important to note that distribution numbers may not accurately reflect naloxone kits in public circulation. Uneven response rates across sites, delays in returning distribution forms, and delays in entering data means that the true count of naloxone kits distributed to the public likely exceeds the reported kits distributed. The authors note that the observed drop in reported kit distribution in 2018 is likely due to a lower return rate, and does not represent a true decrease in naloxone kits distributed to the public. Relatedly, the higher proportion of pharmacy naloxone kit distribution in the Vancouver Coastal Region in the 2018 analytic sample is likely due to low reporting in high volume, low-capacity community sites in the Vancouver region compared to other regions. Operationally, the program has confirmed with high volume sites, which have low reported distribution forms, that kits ordered are being distributed and not held in inventory or being thrown away due to naloxone expiry. The large majority of sites reorder kits as needed on a weekly or monthly basis. Thus, we believe that true distribution is likely much higher than the number of distribution records returned to the BCCDC.

As previously mentioned, careful interpretation of this data is required; the program is low barrier and to ensure anonymity identifiers are not collected from naloxone kit recipients. Therefore, we cannot identify how many unique individuals are accessing or using naloxone kits, or how many kits are distributed and used per individual in BC. Self-reported data is also susceptible to response bias. Stigma around drug use may discourage high-risk individuals from providing information or self-identifying as being at risk of experiencing an overdose, which may in turn bias results.

## Conclusion

Between 2012 and 2018, more than 40,000 take-home naloxone kits have been reported used to reverse an opioid overdose in BC. Community distribution sites are crucial for providing naloxone to people at high risk of overdose, who may be most likely to respond to overdoses in their communities. Pharmacy sites appear to reach a cohort of first-time kit recipients, sustain longer opening and weekend hours, and support access in rural and hard-to-reach areas. Findings from this study highlight the importance of making naloxone available through a variety of distribution sites that are both widely accessible and responsive to the needs of people who require naloxone.

## Supporting information

S1 Appendix Total distribution by Health Authority in BC, from August 31st 2012 to December 31st 2018. (DOCX)

S2 Appendix Total pharmacies in British Columbia by regional Health Authority, 2018. (DOCX)

## References

[1] Ministry of Health. Current Health Topics—Province of British Columbia [Internet]. Province of British Columbia; [cited 2020 Apr 14]. Available from: https://www2.gov.bc.ca/gov/content/health/about-bc-s-health-care-system/office-of-the-provincial-health-officer/current-health-topics

[2] Bethany Lindsay, Canadian Broadcasting Corporation (CBC). B.C. declares public health emergency with 186 cases of COVID-19 and 7 deaths | CBC News [Internet]. CBC. 2020 [cited 2020 Apr 30]. Available from: https://www.cbc.ca/news/canada/british-columbia/b-c-declares-public-health-emergency-with-186-cases-of-covid-19-and-7-deaths-1.5500783

[3] WHO announces COVID-19 outbreak a pandemic [Internet]. World Health Organization; 2020 [cited 2020 May 1]. Available from: http://www.euro.who.int/en/health-topics/health-emergencies/coronavirus-covid-19/news/news/2020/3/who-announces-covid-19-outbreak-a-pandemic

[4] Health Canada. Overview of national data on opioid-related harms and deaths [Internet]. Government of Canada. 2018 [cited 2019 Mar 26]. Available from: https://www.canada.ca/en/health-canada/services/substance-use/problematic-prescription-drug-use/opioids/data-surveillance-research/harms-deaths.html

[5] Coroners Service of BC. Illicit Drug Overdose Deaths in BC [Internet]. Vancouver, BC: Coroners Service of BC, Ministry of Public Safety & Solicitor Genera; 2019 3 [cited 2019 Aug 15] p. 1–21. Available from: https://www2.gov.bc.ca/assets/gov/birth-adoption-death-marriage-and-divorce/deaths/coroners-service/statistical/illicit-drug.pdf

[6] McDonald R, Strang J. Are take-home naloxone programmes effective? Systematic review utilizing application of the Bradford Hill criteria. Addict Abingdon Engl. 2016 3 30;111(7):1177–87. 10.1111/add.13326 PMC5071734 27028542

[7] Kim HK, Nelson LS. Reducing the harm of opioid overdose with the safe use of naloxone: a pharmacologic review. Expert Opin Drug Saf. 2015;14(7):1137–46. 25865597 10.1517/14740338.2015.1037274

[8] Boyer EW. Management of Opioid Analgesic Overdose. N Engl J Med. 2012 7 12;367(2):146–55. 22784117 10.1056/NEJMra1202561 PMC3739053

[9] Amina Moustaqim-Barrette Tara Elton-Marshall, Leece Pamela, Morissette Carole, Rittenbach Katherine, Buxton Jane. Environmental Scan Naloxone Access and Distribution in Canada. 2019 6 30; Available from: https://open.library.ubc.ca/media/stream/pdf/52383/1.0379400/5

[10] BC Centre for Disease Control. Training & Resources [Internet]. Toward the heart. 2020 [cited 2020 May 19]. Available from: https://towardtheheart.com/naloxone-training

[11] About Toward the Heart [Internet]. [cited 2020 Jul 31]. Available from: https://towardtheheart.com/about

[12] British Columbia Centre for Disease Control. Timeline of Community Naloxone in British Columbia [Internet]. British Columbia Centre for Disease Control; 2019. Available from: https://towardtheheart.com/assets/uploads/1574366025Mszqt5dtTudeDPDTmu0VNpJrDrQ2AM0x9UOV7Za.pdf

[13] Pearce LA, Mathany L, Rothon D, Kuo M, Buxton JA. An evaluation of Take Home Naloxone program implementation in British Columbian correctional facilities. Int J Prison Health. 2019;15(1):46–57. 30827160 10.1108/IJPH-12-2017-0058

[14] College of Pharmacists of British Columbia. Naloxone [Internet]. College of Pharmacists of British Columbia. 2016 [cited 2020 Jul 30]. Available from: http://www.bcpharmacists.org/naloxone

[15] Young S, Williams S, Otterstatter M, Lee J, Buxton J. Lessons learned from ramping up a Canadian Take Home Naloxone programme during a public health emergency: a mixed-methods study. BMJ Open. 2019;9(10):e030046. 10.1136/bmjopen-2019-030046 PMC6830612 31662368

[16] Zahra Mamdani, Jane A Buxton. Evaluation of British Columbia’s Take Home Naloxone Program in Community Pharmacies [Internet]. Vancouver, BC: BC Centre for Disease Control; 2019 Available from: https://towardtheheart.com/assets/uploads/1571953664uujS4Wj82uoRg5EBNAHxsi87cDYPGkMRp2TyV9t.pdf

[17] Amina Moustaqim-Barrette, Kristi Papamihali, Jane A Buxton. Take Home Naloxone Report: Review of data to December 2018. [Internet]. Vancouver, BC: BC Centre for Disease Control (BCCDC); 2019 11 p. 33 Available from: https://towardtheheart.com/assets/uploads/1583869759ld78Qe9oxTQg78wGO4woeJ2EXSYrsEEehqzXTMj.pdf

[18] BC Centre for Disease Control. Find A Site [Internet]. Toward the heart. 2020 [cited 2020 Jul 30]. Available from: https://towardtheheart.com/site-finder

[19] Licensed Community Pharmacies Directory [Internet]. College of Pharmacists of British Columbia. [cited 2020 Aug 7]. Available from: http://www.bcpharmacists.org/list-community-pharmacies

[20] Irvine MA, Buxton JA, Otterstatter M, Balshaw R, Gustafson R, Tyndall M, et al Distribution of take-home opioid antagonist kits during a synthetic opioid epidemic in British Columbia, Canada: a modelling study. Lancet Public Health. 2018;3(5):e218–25. 29678561 10.1016/S2468-2667(18)30044-6

[21] Irvine MA, Kuo M, Buxton JA, Balshaw R, Otterstatter M, Macdougall L, et al Modelling the combined impact of interventions in averting deaths during a synthetic-opioid overdose epidemic. Addict Abingdon Engl. 2019;114(9):1602–13. 10.1111/add.14664 PMC6684858 31166621

[22] BC Coroners Service. Illicit Drug Toxicity Deaths in BC: January 1 2020—June 30 2020 [Internet]. British Columbia: Ministry of Public Safety and Solicitor General; 2020 Jul [cited 2020 May 28] p. 22. Available from: https://www2.gov.bc.ca/assets/gov/birth-adoption-death-marriage-and-divorce/deaths/coroners-service/statistical/illicit-drug.pdf

[23] Andrea Woo. Virus measures may be hurting overdose prevention in Vancouver, official says. 2020 Apr 28 [cited 2020 Jun 5]; Available from: https://www.theglobeandmail.com/canada/british-columbia/article-virus-measures-may-be-hurting-overdose-prevention-in-vancouver/

[24] The R Foundation. R: The R Project for Statistical Computing [Internet]. 2019 [cited 2019 Mar 26]. Available from: https://www.r-project.org/

[25] David W. Hosmer, Stanley Lemeshow, Rodney X. Sturdivant. Model‐Building Strategies and Methods for Logistic Regression In: Applied Logistic Regression [Internet]. John Wiley & Sons, Ltd; 2013 [cited 2019 Mar 26]. p. 89–151. Available from: https://onlinelibrary.wiley.com/doi/abs/10.1002/9781118548387.ch4

[26] Zhang Z. Model building strategy for logistic regression: purposeful selection. Ann Transl Med. 2016 3;4(6):111. 27127764 10.21037/atm.2016.02.15 PMC4828741

[27] Choremis B, Campbell T, Tadrous M, Martins D, Antoniou T, Gomes T. The uptake of the pharmacy-dispensed naloxone kit program in Ontario: A population-based study. PloS One. 2019;14(10):e0223589. 31626648 10.1371/journal.pone.0223589 PMC6799925

[28] Leece PN, Hopkins S, Marshall C, Orkin A, Gassanov MA, Shahin RM. Development and implementation of an opioid overdose prevention and response program in Toronto, Ontario. Can J Public Health Rev Can Sante Publique. 2013;104(3):e200–4. 10.17269/cjph.104.3788 PMC6973908 23823882

[29] Freeman LK, Bourque S, Etches N, Goodison K, O’Gorman C, Rittenbach K, et al Alberta’s provincial take-home naloxone program: A multi-sectoral and multi-jurisdictional response to overdose. Can J Public Health Rev Can Sante Publique. 2017;108(4):e398–402. 10.17269/CJPH.108.5989 PMC6972089 29120311

[30] Bozat-Emre S, Marshall SG, Zhong C, Reimer J. At-a-glance—Lessons learned from launching the Manitoba Take-Home Naloxone Program. Apercu—Lecons Tirees Lancement Programme Naloxone Emporter Domic Manit. 2018;38(6):252–5. 10.24095/hpcdp.38.6.06 PMC6034973 29911822

[31] Coroners Service of BC. Illicit Drug Toxicity Deaths in BC, January 1, 2009 –December 31, 2019 [Internet]. Government of British Columbia; 2020. Available from: https://www2.gov.bc.ca/assets/gov/birth-adoption-death-marriage-and-divorce/deaths/coroners-service/statistical/illicit-drug.pdf

[32] Green TC, Case P, Fiske H, Baird J, Cabral S, Burstein D, et al Perpetuating stigma or reducing risk? Perspectives from naloxone consumers and pharmacists on pharmacy-based naloxone in 2 states. J Am Pharm Assoc JAPhA. 2017 4;57(2S):S19–S27.e4. 10.1016/j.japh.2017.01.013 28214219

[33] Zaller ND, Yokell MA, Green TC, Gaggin J, Case P. The feasibility of pharmacy-based naloxone distribution interventions: a qualitative study with injection drug users and pharmacy staff in Rhode Island. Subst Use Misuse. 2013;48(8):590–9. 23750660 10.3109/10826084.2013.793355

[34] Canada H. Exemptions for practitioners and pharmacists prescribing and providing controlled substances, and for patients, during the coronavirus pandemic [Internet]. aem. 2020 [cited 2020 Aug 7]. Available from: https://www.canada.ca/en/health-canada/services/health-concerns/controlled-substances-precursor-chemicals/policy-regulations/policy-documents/section-56-1-class-exemption-patients-pharmacists-practitioners-controlled-substances-covid-19-pandemic.html

[35] BC Centre on Substance Use (BCCSU). COVID-19: Information for Opioid Agonist Treatment Prescribers and Pharmacists [Internet]. British Columbia: BC Centre on Substance Use (BCCSU); 2020 May [cited 2020 Aug 7]. Available from: https://www.bccsu.ca/opioid-use-disorder/

