# Correction: Accessing Take-Home Naloxone in British Columbia and the role of community pharmacies: Results from the analysis of administrative data

**DOI:** 10.1371/journal.pone.0308242

**Published:** 2024-07-30

**Authors:** Amina Moustaqim-Barrette, Kristi Papamihali, Zahra Mamdani, Sierra Williams, Jane A. Buxton

In Table 4, the numbers in the rows under eligibility personally at risk of an overdose and not personally at risk of an overdose are swapped. Please see the correct Table 4 here.

**Table 4:** Adjusted odds ratios and 95% confidence intervals for odds of collecting a kit at pharmacy sites vs other sites.

	Summary characteristics of analytic sample			Multivariate logistic regression	
	Pharmacy (N = 3923)	Other^C^ (N = 53601)	Total (N = 57,524)		
	n (%)	n (%)	n (%*)	AOR (95% CI)	P-value
**Gender**					
Male	2053 (7.0%)	27410 (93.0%)	29458 (51.2%)	1.00	-
Female	1692 (6.7%)	23426 (93.3%)	25123 (43.6%)	0.74 (0.69–0.80)	<0.01
Other^B^	121 (11.8%)	903 (88.2%)	1024 (1.8%)	1.77 (1.44–2.18)	<0.01
Unknown	57 (3.0%)	1862 (97.0%)	1919 (3.3%)	0.39 (0.29–0.52)	<0.01
**Age**					
Under 19	197 (6.4%)	2859 (93.6%)	3056 (5.3%)	1.00	-
19–30	1466 (7.4%)	18346 (92.6%)	19816 (34.4%)	1.41 (1.21–1.66)	<0.01
31–60	1879 (6.1%)	28803 (93.9%)	30689 (53.3%)	1.19 (1.02–1.39)	0.03
Over 60	273 (15.3%)	1512 (84.7%)	1787 (3.1%)	2.43 (1.99–2.97)	<0.01
Unknown	108 (5.0%)	2068 (95.0%)	2176 (3.8%)	1.09 (0.84–1.42)	0.51
**Eligibility**					
Personally at risk of overdose	1566 (4.0%)	37638 (96.0%)	39204 (68.2%)	1.00	-
Not personally at risk of overdose	2357 (12.9%)	15950 (87.1%)	18307 (31.8%)	2.69 (2.50–2.90)	<0.01
**Reason for collecting a kit**					
1^st^ kit	2880 (10.6%)	24195 (89.4%)	27080 (47.1%)	1.00	-
Replacement–other^A^	347 (3.6%)	9232 (96.4%)	9579 (16.7%)	0.42 (0.37–0.47)	<0.01
Replacement–used	696 (3.3%)	20161 (96.7%)	20865 (36.3%)	0.46 (0.42–0.51)	<0.01
**Health Authority**					
Island	532 (3.2%)	15881 (96.8%)	16413 (28.5%)	1.00	-
Fraser	1145 (7.0%)	15279 (93.0%)	16424 (28.6%)	2.79 (2.50–3.10)	<0.01
Interior	923 (7.3%)	11651 (92.7%)	12574 (21.9%)	2.33 (2.08–2.60)	<0.01
Northern	208 (5.3%)	3690 (94.7%)	3898 (6.8%)	1.68 (1.42–1.98)	<0.01
Vancouver Coastal	1115 (13.6%)	7087 (86.4%)	8215 (14.3%)	4.07 (3.65–4.54)	<0.01

Abbreviations: AOR, Adjusted odds ratio; CI, confidence interval*Column percentages

^A^ Take-home naloxone kit was replaced due to a previous kit being lost, stolen, confiscated or expired

^B^ Other genders reported include being trans or gender non-conforming

^C^ Other site types include corrections, pharmacy, post-secondary, other (e.g. housing sites, treatment centres, non-governmental and peer-led organizations)
