# Supplementary material for: Accessing Take-Home Naloxone in British Columbia and the role of community pharmacies: Results from the analysis of administrative data
Source: PLoS One. 2020 Sep 11;15(9):e0238618. doi: 10.1371/journal.pone.0238618 (PMC7485887; doi:10.1371/journal.pone.0238618)
Supplement: S1 Appendix — (DOCX) [file pone.0238618.s001.docx]

| **S1 Appendix – Total Take-Home Naloxone kit distribution by Health Authority in BC, from August 31^st^ 2012 to December 31^st^ 2018.** | | | |
| --- | --- | --- | --- |
|  | **Population^A*^** | **Take-home naloxone kits distributed^B*^** | **Take-home naloxone kits distributed per 1000 people** |
|  | **N (%)** | **N (%)** |  |
| Health Authority |  |  |  |
| Fraser | 1,763,909 (36.9%) | 39,591 (26.4%) | 22.4 |
| Interior | 756,771 (15.8%) | 31,378 (20.9%) | 41.5 |
| Island | 795,852 (16.6%) | 40,092 (26.7%) | 50.4 |
| Northern | 288,152 (6.0%) | 7800 (5.2%) | 27.1 |
| Vancouver Coastal | 1,176,028 (24.6%) | 31,138 (20.8%) | 26.5 |
| **British Columbia** | **4,780,712 (100.0%)** | **149,999 (100.0%)** | **31.4** |
| *^A^Averaged population estimates from 2012 – 2018*  *^B^As explained in this manuscript, distribution records likely represent a significant underestimate of the true number of take-home naloxone kits in circulation in BC*  **Column percentages* | | | |
