# Supplementary material for: Accessing Take-Home Naloxone in British Columbia and the role of community pharmacies: Results from the analysis of administrative data
Source: PLoS One. 2020 Sep 11;15(9):e0238618. doi: 10.1371/journal.pone.0238618 (PMC7485887; doi:10.1371/journal.pone.0238618)
Supplement: S2 Appendix — (DOCX) [file pone.0238618.s002.docx]

| **S2 Appendix – Total pharmacies in British Columbia by regional Health Authority, 2018.** | | | | |
| --- | --- | --- | --- | --- |
|  | **Population^A*^** | **Pharmacies in BC ^*^** | **Participating Pharmacies^*^** | **Participating pharmacies as proportion of total** |
|  | **N (%)** | **N (%)** | **N (%)** | **%** |
| Health Authority |  |  |  |  |
| Fraser | 1,868,894 (37.4%) | 510 (36.7%) | 207 (36.8%) | 40.6% |
| Interior | 816,899 (16.3%) | 220 (15.9%) | 103 (18.3%) | 46.8% |
| Island | 849,447 (17.0%) | 230 (16.6%) | 84 (14.9%) | 36.5% |
| Northern | 282,877 (5.7%) | 76 (5.5%) | 33 (5.9%) | 43.4% |
| Vancouver Coastal | 1,183,053 (23.7%) | 352 (25.2%) | 135 (24.0%) | 38.4% |
| **British Columbia** | **5,001,170 (100.0%)** | **1388 (100.0%)** | **562 (100%)** | **40.5%** |
| *^A^ Population estimate for 2018*  **Column percentages* | | | | |
